# H-marker via bronchoscopy under LungPro navigation combined with cone-beam computed tomography for locating multiple pulmonary ground-glass nodules: A case report and literature review

**DOI:** 10.1097/MD.0000000000039805

**Published:** 2024-09-20

**Authors:** Wanlan Fang, Jisong Zhang, Enguo Chen, Kejing Ying

**Affiliations:** a Department of Respiratory and Critical Care Medicine, Regional Medical Center for National Institute of Respiratory Diseases, Sir-Run-Run-Shaw Hospital, School of Medicine, Zhejiang University, Hangzhou, China; b Department of Respiratory and Critical Care Medicine, Zhejiang Deqing People’s Hospital, Deqing, China.

**Keywords:** bronchoscopy, case report, cone-beam computed tomography, implanted fiducials, pulmonary ground-glass nodule, surgical navigation systems

## Abstract

**Rationale:**

Pulmonary ground-glass nodules (GGNs) pose challenges in intraoperative localization due to their primarily nonsolid composition. This report highlights a novel approach using H-marker deployment guided by LungPro navigation combined with cone-beam computed tomography (CBCT) for precise localization of multiple GGNs.

**Patient concerns:**

A 55-year-old female patient presented at Sir-Run-Run-Shaw Hospital, Zhejiang University School of Medicine, in June 2021, requiring thoracoscopic surgery for the management of multiple GGNs in her right lung. She had a recent history of thoracoscopic wedge resection for a lesion in her lower left lung 3 months prior.

**Diagnoses:**

Computed tomography scans revealed the presence of 3 mixed GGNs in the right lung, with further confirmation identifying these as solitary pulmonary nodules, necessitating surgical intervention.

**Interventions:**

The patient underwent thoracoscopic surgery, during which the multiple nodules in her right lung were precisely localized utilizing an H-marker implanted bronchoscopically under the guidance of LungPro navigation technology, with CBCT providing additional confirmation of nodule positioning. This innovative combination of technologies facilitated accurate targeting of the lesions.

**Outcomes:**

Postoperative histopathological analysis confirmed the nodules to be microinvasive adenocarcinomas. Radiographic examination with chest X-rays demonstrated satisfactory lung expansion, indicating effective lung function preservation following the procedure. Follow-up assessments have shown no evidence of tumor recurrence, suggesting successful treatment.

**Lessons:**

The employment of H-marker implantation guided by the LungPro navigation system with CBCT confirmation presents a feasible and efficacious strategy for localizing multiple pulmonary GGNs. To further validate its clinical utility and safety, large-scale, multicenter, prospective studies are warranted. This approach holds promise in enhancing the precision and outcomes of surgeries involving GGNs.

## 1. Introduction

Lung cancer is the leading cause of cancer incidence and mortality worldwide.^[1]^ Surgical resection is the first choice for treating pulmonary nodules^[2]^; yet, small nodules are often difficult to see on the lung surface. The success rate of intraoperatively locating lesions using manual palpation or instrument exploration is only 30%.^[3]^ Ground-glass nodules (GGNs) have few solid components, and intraoperative localization is even more difficult.^[4]^ Therefore, accurate preoperative localization is vital for the accurate resection of lesions and maximum protection of pulmonary function.

Common preoperative auxiliary localization technology for pulmonary nodules includes computed tomography (CT)–guided percutaneous puncture localization,^[5]^ electromagnetic navigation bronchoscopy (ENB)–guided localization,^[6]^ and CT virtual 3-dimensional (3D) auxiliary localization.^[7,8]^ Nevertheless, these methods are associated with some risks and have certain limitations. The LungPoint augmented reality navigation system and the LungPro whole-lung diagnosis and treatment navigation system have been widely used. The navigation system displays 2 main images during bronchoscopy, that is, a real-time bronchoscopy video and virtual bronchoscopy animation. Navigational bronchoscopy is a painless, noninvasive, safe, and rapid tool.^[9]^ Herein, we reported a single case of a patient with multiple pulmonary GGNs located using an H-marker via bronchoscopy under LungPro navigation combined with cone-beam CT (CBCT) confirmation.

## 2. Case presentation

**Figure 1.**
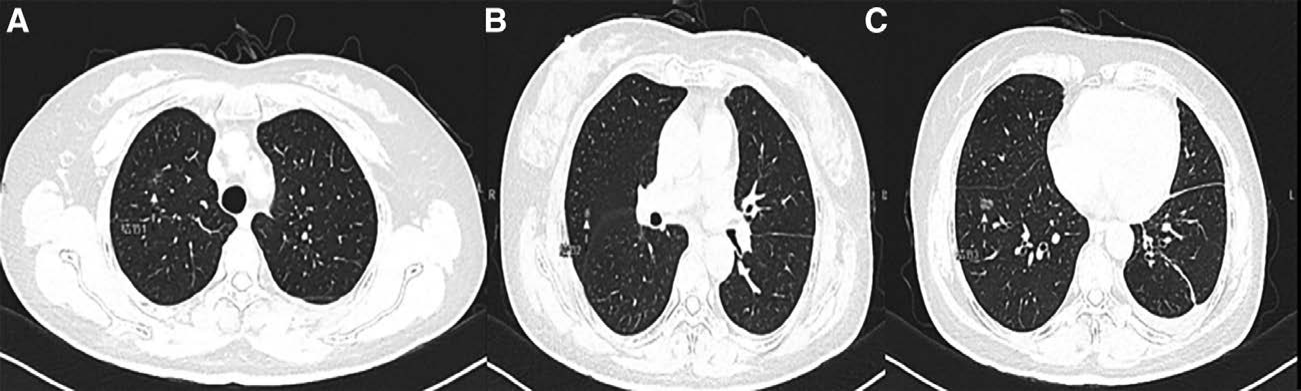
CT images before surgery. (A) Nodule 1 in the apical segment of the upper right leaflet. (B) Nodule 2 in the anterior segment of the right upper lobe. (C) Nodule 3 in the anterior basal segment of the right lower lobe. CT = computed tomography.

**Figure 2.**
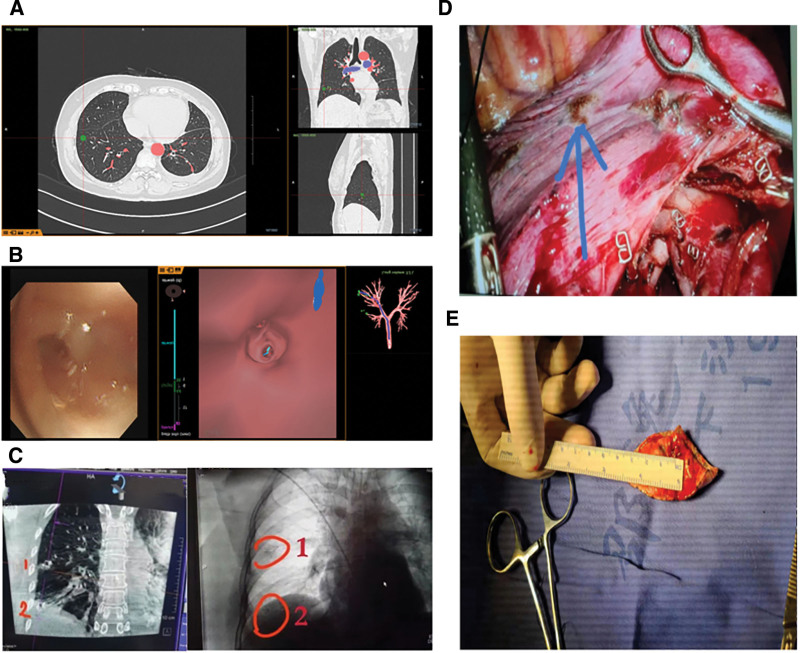
Surgical flowchart. (A) 3D reconstruction using thin-slice chest CT data. (B) Intraoperative guidance under the LungPro navigation system of the anterior segment of the right upper lobe. (C) H-marker positioning point; position confirmed by CBCT. (D) Exploration under the endoscope; the H-marker was successfully detected. (E) Pulmonary segment resection specimen; it can be seen that the gray-white cancerous nodules have been accurately resected. 3D = 3 dimensional, CBCT = cone-beam computed tomography, CT = computed tomography.

A 55-year-old woman was scheduled for thoracoscopic surgery at the Sir-Run-Run-Shaw Hospital affiliated with Zhejiang University School of Medicine in June 2021 due to multiple pulmonary GGNs in her right lung. The patient had multiple lung nodules for over 3 years and underwent thoracoscopic wedge resection of the lower left lung 3 months ago. Postoperative pathology showed microinvasive adenocarcinoma; postoperative reexamination showed that carcinoembryonic antigen was 1.22 ng/mL, neuron-specific enolase was 15.6 ng/mL, cytokeratin 19 fragment was 0.97 µg/L, and squamous cell carcinoma antigen was 0.30 ng/mL. Postoperative CT showed 3 mixed GGNs in the apical segment of the upper right lung, the anterior segment of the upper right lung, and the anterior basal segment of the lower right lung (Fig. 1). Solitary pulmonary nodules were confirmed, and thoracoscopic surgery was performed. Before tracheoscopy, 3D reconstruction using thin-slice chest CT data was performed to plan and calculate the path of navigation tracheoscopy. Under the guidance of the LungPro navigation system, the tracheoscope reached the preset target near the lesion in 3D reconstruction (Fig. 2A). The H-marker implant was directly inserted into the working channel of the tracheoscope and placed at each of the nodule sites for localization (Fig. 2B). After release, CBCT was used to confirm the positioning of the H-markers in real time (Fig. 2C). Then, video-assisted thoracoscopic surgery was performed, and nodules were resected (Fig. 2D and 2E).

Postoperative pathology showed that the nodules in the apical segment of the upper right lobe and the anterior segment of the upper right lobe were microinvasive adenocarcinomas, while the nodule in the anterior basal segment of the lower right lobe was adenocarcinoma. Postoperative chest X-ray showed good lung recruitment and no tumor recurrence at the most recent follow-up.

## 3. Discussion and Conclusions

The present study introduced a case with multiple ground-glass pulmonary nodules located through the H-marker via bronchoscopy under LungPro navigation combined with CBCT confirmation. This study suggested a new approach for the location of pulmonary nodules during thoracoscopic surgery.

Regarding the selection of positioning materials, most studies reporting on the localization of pulmonary nodules using bronchoscopy (Table S1, Supplemental Digital Content, http://links.lww.com/MD/N621; illustrates the summary of the research results on transbronchoscopic navigation and localization reported in the literature) injected liquid dyes via ENB. The most common liquid materials included methylbenzene, medical glue, indocyanine green, and lipiodol. Localization failure was primarily related to the diffusion of the liquid dye. There are few reports on the placement of metal markers with navigation bronchoscopy used to locate pulmonary nodules. In their study, Toba et al^[10]^ marked 63 pulmonary nodules in 58 patients via transbronchial coil placement. A metallic coil was installed in the bronchus nearest to the lesion under CT fluoroscopic guidance by ultrathin bronchoscopy. A virtual bronchoscopy navigation (VBN) system was used in 14 cases. The results showed that the average time required for localization was 38.9 (15–120) minutes, the localization success rate was 98.4%, pneumothorax occurred in 1.7% of cases, and there were no fatal complications. The duration of the examination and CT examination in the 14 cases in the VBN group was significantly reduced compared with the common bronchoscopy group. Sato et al^[11]^ performed a multicenter, prospective, single-arm study in 64 patients with 65 pulmonary nodules using the virtual-assisted bronchoscopy localization technique (VAL-MAP 2.0) with dye labeling and coil placement in a distal bronchus. The specific method involved performing 3D reconstruction using preoperative chest CT data and delineating the virtual resection range. Under the guidance of the virtual image, the bronchoscope was used to inject indigo carmine into each target bronchus to determine the peripheral resection range; then, a coil was inserted into the target lesion through the bronchoscope. The coil determined the depth of resection. After the positioning was completed, CT was performed to confirm the marker’s location. The CT images obtained after VAL-MAP were used for 3D reconstruction to provide a reference in the final surgery, and the surgery was performed 1 to 2 days after marker placement. The results showed that the success rate of dye labeling was 88.3%, the success rate of coil placement was 82.4%, 6.2% of patients had mild pneumothorax, 6.2% had a fever, and the success rate of surgical resection was 98.5%.

Chen et al^[12]^ injected a lipiodol mixture to simulate 12 pulmonary nodules in 3 porcine models. Two coils were placed near each lesion via VBN combined with intraoperative fluoroscopy 1 week later. CT was performed 1 day, 1 week, 2 weeks, and 4 weeks after positioning. At 4 weeks, 87.5% of the coils showed 0- to 5-mm displacement, 8.33% showed 5- to 10-mm displacement, and 4.17% showed >10-mm displacement. No complications, including pneumothorax and hemorrhage, were observed after positioning or during follow-up after thoracoscopy. The simulated lesions were surgically removed under fluoroscopy 5 weeks after positioning, and the success rate of surgical resection was 100%. Following this result, an exploratory clinical study was performed in 3 patients with 3 pure ground-glass pulmonary nodules using 1 coil near each target lesion under ENB and CBCT guidance. No complications, such as pneumothorax, were observed. Video-assisted thoracoscopic surgery was performed immediately afterward, and the success rate of surgical resection was 100%.

The positioning material used in our patients, the H-marker, is a special 1-time-use pulmonary surgical marker specifically designed for navigation bronchoscopy. An appropriate radial support force was used to ensure that no shifting from the target position occurred during the anchoring of the marker. H-markers have a moderate hardness, which is convenient for identification during exploration in thoracic surgery. The literature review revealed no reports of special pulmonary nodule localization markers for navigation tracheoscopy. Because nodules of early-stage lung cancer are generally located in the periphery and are small in diameter,^[13]^ localization via bronchoscopy often requires navigation and a guidance system to ensure accurate marker positioning. The most used guidance method is bronchoscopy navigation. Current mainstream navigation systems include VBN and ENB. The basic working principle of these systems is to import the patient’s high-resolution spiral chest CT scan into the navigation system in digital imaging and communication in medicine format and reconstruct it into 3D tracheobronchial images and pulmonary vascular images. The path to peripheral lung lesions is calculated by the navigation system and matched with the bronchoscopy image in real time to allow the surgeon to accurately reach the peripheral lesions. VBN systems have no real-time navigation function and must be combined with X-ray fluoroscopy, CBCT, or radial endobronchial ultrasound to confirm when the lesion is reached.^[14]^ Based on virtual imaging, ENB systems are equipped with electromagnetic probes for real-time guidance. During the surgery, the patient is in a 3D magnetic field. The electromagnetic probe inserted into the bronchoscope matches the actual and virtual images in real time and guides the electromagnetic probe to the lesion site to perform needle aspiration, brushing, biopsy, or dye injection for localization.^[15,16]^ ENB systems are equipped with a magnetic positioning probe that achieves relative real-time positioning but is limited by the high cost of magnetic positioning consumables.^[17]^

Eberhardt et al^[18]^ used the LungPoint augmented reality navigation system for the transbronchial biopsy of 25 pulmonary nodules in 25 patients. Navigation showed that the bronchial branches of all pulmonary nodules on the virtual bronchoscopy image were in good agreement with the actual branches, and 80% of the patients were definitively diagnosed without life-threatening complications. The LungPro whole-lung diagnosis and treatment navigation system also avoids blood vessels to allow bronchoscopic transparenchymal nodule access surgery to be performed and establishes a tunnel leading directly to lesions outside the airway, which is helpful for the diagnosis and treatment of pulmonary nodules outside the airway and provides whole-lung access.^[19]^ Compared with ENB systems, the LungPro whole-lung diagnosis and treatment navigation system does not require any positioning consumables during the entire process, and the economic cost for the patients is significantly reduced. Compared with VBN, the LungPro system provides a certain degree of real-time matching, effectively solving the limitations of VBN in inspection. However, most of the recent studies focused on the puncture biopsy of peripheral pulmonary nodules under LungPro navigation via bronchoscopy, and there has been no relevant research on the preoperative localization of pulmonary nodules using bronchoscopy under LungPro navigation.

CBCT is a medical imaging modality that combines a C-arm digital flat panel detector angiography system with an improved CT reconstruction technology. It enables both 3D angiography reconstruction and CT imaging, generating CT-like images of the soft tissue. The main difference between traditional CT and CBCT is that CBCT uses high-resolution 2-dimensional detectors instead of multiple 1-dimensional detectors to obtain information. The level of radiation is low, and the operation is simple. CBCT can be used to monitor the location of lesions in real time during interventional surgery to achieve the best diagnosis and treatment results.^[20,21]^ As a real-time on-site extrathoracic navigation confirmation method to assist bronchoscopy, CBCT has been reported in many studies.^[22,23]^ Verhoeven et al^[24]^ applied CBCT and ENB guidance during the puncture biopsy of 105 pulmonary nodules in 87 patients. The results showed a 76.3% and 52.2% navigation success rate for the CBCT and ENB methods, respectively. Adding CBCT to the ENB method increased the navigation success rate to 87.5% for each lesion. A phantom study of CBCT and bronchoscopy navigation to peripheral nodules by Hohenforst-Schmidt et al^[20]^ revealed very low body radiation doses of 0.98 to 1.15 mSv from a single CBCT session. Therefore, CBCT for the biopsy and localization of pulmonary nodules can provide navigation guidance for localization, biopsy, and pathological confirmation, and the combined use of CBCT with navigation bronchoscopy is expected to improve the success rate of localization and biopsy.

In conclusion, H-marker implantation using a bronchoscope under LungPro navigation and CBCT may be used to locate multiple GGNs. Multicenter prospective studies with large sample sizes are needed to validate and enhance the applicability of the approach.

## Author contributions

**Formal analysis:** Wanlan Fang, Enguo Chen, Kejing Ying.

**Investigation:** Wanlan Fang, Jisong Zhang.

**Methodology:** Wanlan Fang, Jisong Zhang, Enguo Chen.

**Visualization:** Wanlan Fang.

**Writing – original draft:** Wanlan Fang, Jisong Zhang, Enguo Chen, Kejing Ying.

**Writing – review & editing:** Wanlan Fang, Jisong Zhang, Enguo Chen, Kejing Ying.

**Data curation:** Jisong Zhang, Kejing Ying.

**Validation:** Jisong Zhang, Enguo Chen, Kejing Ying.

**Conceptualization:** Enguo Chen.

**Project administration:** Enguo Chen.

**Funding acquisition:** Kejing Ying.

## Supplementary Material



## References

[1] Sung H Ferlay J Siegel RL. Global cancer statistics 2020: GLOBOCAN estimates of incidence and mortality worldwide for 36 cancers in 185 countries. CA Cancer J Clin. 2021;71:209–49. 33538338 10.3322/caac.21660

[2] NCCN Clinical Practice Guidelines in Oncology (NCCN Guidelines). Non-Small Cell Lung Cancer. Version 3.2023. Fort Washington: National Comprehensive Cancer Network. 2023.

[3] Barmin V Sadovnichy V Sokolov M Pikin O Amiraliev A. An original device for intraoperative detection of small indeterminate nodulesdagger. Eur J Cardiothorac Surg. 2014;46:1027–31. 24740934 10.1093/ejcts/ezu161

[4] Kim YT. Management of ground-glass nodules: when and how to operate? Cancers (Basel). 2022;14:715. 35158981 10.3390/cancers14030715 PMC8833330

[5] McDermott S Fintelmann FJ Bierhals AJ. Image-guided preoperative localization of pulmonary nodules for video-assisted and robotically assisted surgery. Radiographics. 2019;39:1264–79. 31419188 10.1148/rg.2019180183

[6] Wang G Lin Y Zheng L. A new method for accurately localizing and resecting pulmonary nodules. J Thorac Dis. 2020;12:4973–84. 33145071 10.21037/jtd-20-2089 PMC7578447

[7] Zhang L Wang L Kadeer X. AME Thoracic Surgery Collaborative Group. Accuracy of a 3-dimensionally printed navigational template for localizing small pulmonary nodules: a noninferiority randomized clinical trial. JAMA Surg. 2019;154:295–303. 30586136 10.1001/jamasurg.2018.4872 PMC6484816

[8] Fu R Chai YF Zhang JT. Three-dimensional printed navigational template for localizing small pulmonary nodules: a case-controlled study. Thorac Cancer. 2020;11:2690–7. 32686309 10.1111/1759-7714.13550 PMC7471015

[9] Yanagiya M Kawahara T Ueda K Yoshida D Yamaguchi H Sato M. A meta-analysis of preoperative bronchoscopic marking for pulmonary nodules. Eur J Cardiothorac Surg. 2020;58:40–50. 32563193 10.1093/ejcts/ezaa050

[10] Toba H Kondo K Miyoshi T. Fluoroscopy-assisted thoracoscopic resection after computed tomography-guided bronchoscopic metallic coil marking for small peripheral pulmonary lesions. Eur J Cardiothorac Surg. 2013;44:e126–32. 23598353 10.1093/ejcts/ezt220

[11] Sato M Kobayashi M Sakamoto J. The role of virtual-assisted lung mapping 2.0 combining microcoils and dye marks in deep lung resection. J Thorac Cardiovasc Surg. 2022;164:243–51.e5. 34654560 10.1016/j.jtcvs.2021.09.016

[12] Chen J Pan X Gu C. The feasibility of navigation bronchoscopy-guided pulmonary microcoil localization of small pulmonary nodules prior to thoracoscopic surgery. Transl Lung Cancer Res. 2020;9:2380–90. 33489800 10.21037/tlcr-20-1206 PMC7815366

[13] Yu G Liu X Li Y. The nomograms for predicting overall and cancer-specific survival in elderly patients with early-stage lung cancer: a population-based study using SEER database. Front Public Health. 2022;10:946299. 36159305 10.3389/fpubh.2022.946299 PMC9493218

[14] Wang LL Li XY Wang SY. Application of transbronchial navigation system in the treatment of peripheral pulmonary diseases. Chin J Tuberculosis Resp Dis. 2019;42:140–4. 10.3760/cma.j.issn.1001-0939.2019.02.014 30704190

[15] Schwarz Y. Electromagnetic navigation. Clin Chest Med. 2010;31:65–73, Table of contents. 20172433 10.1016/j.ccm.2009.08.005

[16] Chen Y Li SY. New progress in clinical application of electromagnetic navigation bronchoscopy. Chin J Tuberculosis Resp Dis. 2013;36:6–8. 23537533

[17] Dale CR Madtes DK Fan VS Gorden JA Veenstra DL. Navigational bronchoscopy with biopsy versus computed tomography-guided biopsy for the diagnosis of a solitary pulmonary nodule: a cost-consequences analysis. J Bronchology Interv Pulmonol. 2012;19:294–303. 23207529 10.1097/LBR.0b013e318272157d PMC3611239

[18] Eberhardt R Kahn N Gompelmann D Schumann M Heussel CP Herth FJ. LungPoint--a new approach to peripheral lesions. J Thorac Oncol. 2010;5:1559–63. 20802352 10.1097/JTO.0b013e3181e8b308

[19] Herth FJ Eberhardt R Sterman D Silvestri GA Hoffmann H Shah PL. Bronchoscopic transparenchymal nodule access (BTPNA): first in human trial of a novel procedure for sampling solitary pulmonary nodules. Thorax. 2015;70:326–32. 25746631 10.1136/thoraxjnl-2014-206211

[20] Hohenforst-Schmidt W Zarogoulidis P Vogl T. Cone beam computertomography (CBCT) in interventional chest medicine - high feasibility for endobronchial realtime navigation. J Cancer. 2014;5:231–41. 24665347 10.7150/jca.8834 PMC3963080

[21] Bai M Liu B Mu H Liu X Jiang Y. The comparison of radiation dose between C-arm flat-detector CT (DynaCT) and multi-slice CT (MSCT): a phantom study. Eur J Radiol. 2012;81:3577–80. 21963617 10.1016/j.ejrad.2011.09.006

[22] Sobieszczyk MJ Yuan Z Li W Krimsky W. Biopsy of peripheral lung nodules utilizing cone beam computer tomography with and without trans bronchial access tool: a retrospective analysis. J Thorac Dis. 2018;10:5953–9. 30505506 10.21037/jtd.2018.09.16 PMC6236162

[23] Piro R Fontana M Casalini E. Cone beam CT augmented fluoroscopy allows safe and efficient diagnosis of a difficult lung nodule. BMC Pulm Med. 2021;21:327. 34670551 10.1186/s12890-021-01697-y PMC8527755

[24] Verhoeven RLJ Futterer JJ Hoefsloot W van der Heijden E. Cone-beam CT image guidance with and without electromagnetic navigation bronchoscopy for biopsy of peripheral pulmonary lesions. J Bronchology Interv Pulmonol. 2021;28:60–9. 32649327 10.1097/LBR.0000000000000697 PMC7742216

