# Supplementary material for: H-marker via bronchoscopy under LungPro navigation combined with cone-beam computed tomography for locating multiple pulmonary ground-glass nodules: A case report and literature review
Source: Medicine (Baltimore). 2024 Sep 20;103(38):e39805. doi: 10.1097/MD.0000000000039805 (PMC11419563; doi:10.1097/MD.0000000000039805)
Supplement: Supplementary file 1 [file medi-103-e39805-s001.docx]

**Table S1.** Summary of the research results on transbronchoscopic navigation and localization reported in the literature

| Author | Year | Patient number | Nodule number | Positioning method | Positioning time (min) | Success rate of positioning (%) | Interval between positioning and surgery | Complication rate of positioning (%) | Success rate of surgical resection (%) |
| --- | --- | --- | --- | --- | --- | --- | --- | --- | --- |
| Sakamoto et al. (25) | 2001 | 6 | 6 | Injection of indigo carmine through the bronchoscope | 30 | 100 | 1 day (5 cases), 3 days (1 case) | 0 | 100 |
| Endo et al. (26) | 2004 | 17 | 17 | Injection of indigo carmine through a bronchoscope, confirmation by CT | 35 | 100 | 1 day (16 cases), 3 days (1 case) | 0 | 100 |
| Toba et al. (10) | 2013 | 58 | 63 | Coil marking through the bronchoscope, confirmation by CT (14 cases were marked under VBN guidance) | 38.9 | 98.4 | 5.6 (0-30) days | 1.7 | 100 |
| Krimsky et al. (27) | 2014 | 21 | 21 | 10 cases, injection with indigo carmine through ENB; 11 cases, injection with MB | Not mentioned | 81 | Surgery immediately after positioning | 0 | 100 |
| Sato et al. (28) | 2014 | 30 | 37 | VAL-MAP | 5-15 | 92.6 | 1 day (7 cases),  23 cases were on the same day | 3.3 | 100 |
| Tay et al. (29) | 2016 | 6 | 6 | 5 cases, injection with indigo carmine through ENB; 1 case, injection with MB | Not mentioned | 100 | 1 day | 0 | 100 |
| Awais et al. (30) | 2016 | 29 | 33 | ENB-guided MB marking | 9.7 | 100 | Surgery immediately after positioning | 0 | 100 |
| Abbas et al. (31) | 2017 | 51 | 54 | Injection of liquid marker through ENB (21 cases with MB, 30 cases with MB+ICG) | 29.1 | 98.1 | Surgery immediately after positioning | 0 | 100 |
| Luo et al. (32) | 2017 | 24 | 30 | Injection of fibrin sealant mixed with MB through ENB | 18 | 100 | 22.1 (15-40) | 4.2% | 100 |
| Muñoz-Largacha et al. (33) | 2017 | 17 | 19 | ENB-guided MB marking | Not mentioned | 79 | Surgery immediately after positioning | 0 | 100 |
| Hachey et al. (34) | 2017 | 12 | 14 | Injection of MB through NB | 34 | 100 | Surgery immediately after positioning | 0 | 100 |
| Pupovac et al. (35) | 2017 | 15 | 15 | ENB-guided MB marking | 22 | 6.6 | Surgery immediately after positioning | 0 | 93.3 |
| Anayama et al. (36) | 2018 | 22 | 28 | Injection of ICG/iopamidol mixture through VBN | ＜30 | 90.9 | 1 day | 0 | 100 |
| Xu et al. (37) | 2018 | 60 | 60 | R-EBUS-guided MB marking through an ultrathin bronchoscope | Not mentioned | 91.7 | Surgery immediately after positioning | 0 | 8 cases of conversion to thoracotomy |
| Yanagiya et al. (38) | 2019 | 14 | 17 | VAL-MAP;  injection position (supine, 7/lateral, 10) | Not mentioned | Supine, 57/lateral, 88 | 1 day | 14.2 | Supine, 100/lateral, 78) |
| Hyun et al. (39) | 2019 | 20 | 24 | ENB-guided indigo carmine marking | Median navigation time 4.5, other not mentioned | 95.8 | Surgery immediately after positioning | 0 | 95.8 |
| Song et al. (40) | 2019 | 22 | 22 | ENB-guided vectorial localization | 17.5±4.2 | 100 | Surgery immediately after positioning | 0 | 100 |
| Qian et al. (41) | 2019 | 15 | 15 | ENB-guided MB marking | 26.7±5.3 | 100 | Surgery immediately after positioning | 0 | 100 |
| Wang et al. (42) | 2020 | 16 | 20 | ENB-guided ICG marking | 12.06±2.74 | 100 | Surgery immediately after positioning | 0 | 100 |
| Gu et al. (43) | 2020 | 20 | Not mentioned | ENB-guided MB marking | Not mentioned | 100 | Surgery immediately after positioning | 0 | 100 |
| Tian et al. (44) | 2020 | 52 | 52 | ENB-guided MB marking | 21.29 ±4.0 | 100 | Surgery immediately after positioning | 0 | 100 |
| Chenet al. (12) | 2020 | 3 pig models;/ 3 patients with pulmonary nodules | 12 simulated lung lesions; /3 pulmonary nodules | Placement of 2 microcoils in every simulated lung lesion under VBN, confirmed with fluoroscopy;/placement of microcoils around nodule under ENB guidance, confirmed with CBCT | Not mentioned | 100/100 | 5 weeks/Surgery immediately after positioning | 0/0 | 100/100 |
| Sato et al. (11) | 2021 | 64 | 65 | Combined micro coil positioning based on VAL-MAP | Not  mentioned | Dye  Marks, 88.3; micro coils, 82.4 | 0-2 days | 12.4 | 98.5 |
| Mariolo et al. (45) | 2021 | 48 | 48 | ENB-guided MB marking | 25 | 94 | Surgery immediately after positioning | 0 | 100 |
| Song et al. (46) | 2021 | 134 | 164 | ENB-guided dye marking | 29.4 ± 15.7 | 94.5 | Not  mentioned | 0 | Not  mentioned |

R-EBUS, radial endobronchial ultrasound; VBN, virtual bronchoscopy navigation; MB, methylene blue; VAL-MAP, virtual-assisted lung mapping; ENB, electromagnetic navigation bronchoscopy; ICG, indocyanine green; CBCT, cone-beam computed tomography.
